# Role of Heat-Shock Proteins in Cellular Function and in the Biology of Fungi

**DOI:** 10.1155/2015/132635

**Published:** 2015-12-31

**Authors:** Shraddha Tiwari, Raman Thakur, Jata Shankar

**Affiliations:** Department of Biotechnology and Bioinformatics, Jaypee University of Information Technology, Solan, Himachal Pradesh 173234, India

## Abstract

Stress (biotic or abiotic) is an unfavourable condition for an organism including fungus. To overcome stress, organism expresses heat-shock proteins (Hsps) or chaperons to perform biological function. Hsps are involved in various routine biological processes such as transcription, translation and posttranslational modifications, protein folding, and aggregation and disaggregation of proteins. Thus, it is important to understand holistic role of Hsps in response to stress and other biological conditions in fungi. Hsp104, Hsp70, and Hsp40 are found predominant in replication and Hsp90 is found in transcriptional and posttranscriptional process. Hsp90 and Hsp70 in combination or alone play a major role in morphogenesis and dimorphism. Heat stress in fungi expresses Hsp60, Hsp90, Hsp104, Hsp30, and Hsp10 proteins, whereas expression of Hsp12 protein was observed in response to cold stress. Hsp30, Hsp70, and Hsp90 proteins showed expression in response to pH stress. Osmotic stress is controlled by small heat-shock proteins and Hsp60. Expression of Hsp104 is observed under high pressure conditions. Out of these heat-shock proteins, Hsp90 has been predicted as a potential antifungal target due to its role in morphogenesis. Thus, current review focuses on role of Hsps in fungi during morphogenesis and various stress conditions (temperature, pH, and osmotic pressure) and in antifungal drug tolerance.

## 1. Introduction

Kingdom Fungi encompass a diverse taxonomy involving filamentous and nonfilamentous fungus, which can be classified on the basis of diversity, morphology, growth and development, reproduction, evolution, ability of causing infection, and toxigenicity [1, 2]. Throughout the evolution, fungi have developed diverse mode of reproduction and ability to adapt to their environment [3]. Mode of feeding is absorption in fungi, for which they adhere or grow within the substrate in the form of hyphae. During adverse conditions, filamentous growth takes place to allow easier nutrient diffusion by providing large surface area of hyphae. Generally, fungi require warm and humid conditions for growth. Decrease in temperature causes fungal dormancy (spores are resistant to cold), while increasing temperature leads to degradation of fungi [1]. Thus, temperature initiates stress responses in fungi, which can be either heat-shock or cold-shock affecting the life cycle and cellular processes. Optimum temperature for growth of various fungi (*Histoplasma capsulatum, Aspergillus fumigatus, *and* Cryptococcus neoformans*) is around 37°C [4, 5]. Increase in temperature generally causes attenuation and ultimately leads to death of the organism [6, 7]. Fungus such as* Saccharomyces cerevisiae* can grow at higher temperature (41°C) [8]. In dimorphic fungi (*H. capsulatum*), morphology and temperature are linked with each other which converts from filamentous to yeast form at elevated temperature and vice versa [9].

Stress is a critical factor and plays a key role in functional characteristics of fungi. Protein denaturation has been reported in stress and during modulation in temperature, which causes native misfolding of protein and protein aggregation ultimately leading to the loss of biological functions and also leads to cell apoptosis [10]. The stress related changes are responded by a set of proteins, which facilitate survival of the organism. The family of these proteins is termed as Hsps. Hsps are found ubiquitously in a cell (cytosol, mitochondria, endoplasmic reticulum, nucleus, and cell membrane) [11]. Major role of Hsps involves the cell cycle progression, replication, and transcriptional and posttranslational processes such as protein folding, stability, transportation, and degradation and they are also reported in the activation of many key signal transducers in fungi [11, 12]. Hsps are highly conserved biomolecules which are constitutively expressed and upregulated in response to various stress conditions (biotic and abiotic) [13]. It is also suggested that Hsp plays an important role in homeostasis stress response.

**Figure 1:**
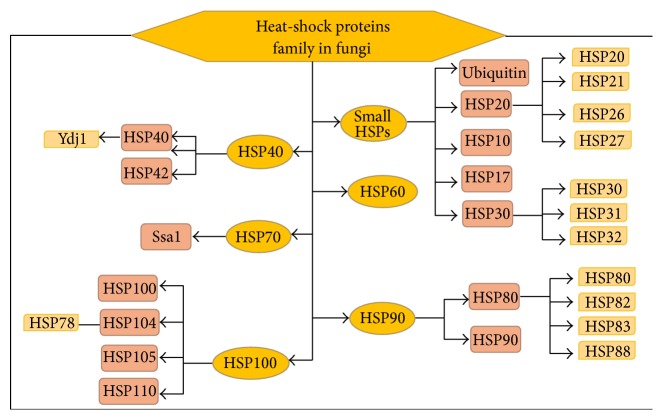
Heat-shock proteins family in fungi categorised on the basis of molecular mass and functional role. Subfamily and classes are derived from previous reviews [20, 23–26].

In protein related disorders, Hsps act as disease suppressor by acting as catalytic polypeptide unfolding isomerase and refolding the mismatched or aggregated proteins [14]. Hsps are categorised on the basis of their molecular weight. Hsps are involved in many regulatory pathways and behave as molecular chaperons for other cellular proteins [11]. The Hsps range in molecular weight from 15 to 110 kDa and are divided into groups based on both size and function [11, 15]. Hsps are divided into several families based on their molecular mass: 100, 90, 70, and 60 kDa [16]. Also, with low molecular mass of 12–43 kDa they are known as small Hsps. Small Hsps contain 80–100 amino acids conserved site at the C*-*terminus and a *α*/*β*-crystalline domain [17]. In addition, it is believed that there exists another class of low-molecular-weight Hsp, ubiquitin (8 kDa), characteristic of eukaryotic organisms [18]. Hsps are classified based on their functions: chaperones (Hsps 70 and 60), proteins with catalytic activity (proteases, Hsp100, ubiquitin, and tyrosine phosphatase), and proteins with an obscure function (*α*-crystalline and secreted glycoproteins) [19]. Different Hsps in fungi on the basis of their molecular weight, cellular localization, and functional characterization are illustrated in Table 1 and fungal Hsps families are presented in Figure 1. Hsps are induced by two mechanisms in fungi, specific mechanism and general mechanism. The former is induced by temperature stress and the latter by other stresses such as pH, oxidative stress, osmotic stress, starvation, or antifungal stress [20]. Thus, we reviewed the role of different Hsps involved in fungal biology and their role in both optimal growth conditions and stress responses. Studies have shown that the predominant Hsps of fungal kingdom are Hsp90, Hsp70, and Hsp20–40, which play crucial role in morphogenetic changes, stress adaptation, and antifungal resistance [21, 22].

This review summarizes the role of Hsps involved in functional characteristics of fungi (*S. cerevisiae*,* C. albicans*,* P. brasiliensis*, and* A. fumigatus*) that includes morphogenesis (conidiation and dimorphism) and various stress responses such as heat stress, pH (acidity/basicity), osmolarity, and antifungal tolerance studies of fungi.

## 2. Role of Hsp90 and Hsp70 in Fungal Morphogenesis

Generally asexual reproduction of fungi involves four different stages starting with dormant conidia which convert into vegetative hyphae after few hours and grow to form a network of hyphae called mycelia. Mycelium leads to the formation of aerial hyphae which produces conidia [27]. The Hsps participate in the morphogenesis of fungi and play a major role in the replication, transcription, posttranscriptional process, translation, posttranslational processes, and the activation of signalling pathways. In yeasts (*S. cerevisiae* and* C. albicans*), Hsp104 in association with Hsp40 and Hsp70 helps in reactivation and aggregation of denatured protein, by providing disaggregated protein to Hsp40 and Hsp70 as a substrate [28, 29]. In addition to these functions, Hsp104 is also involved in replication of yeast prions, for example, PIN1 and URE3 [30]. Expression of Hsp104 and Hsp70 is regulated by Hsp-Hsf (heat-shock factor) interaction which can be stimulated by heat stress in yeast [31]. Cdc37p, cochaperon of Hsp90 in* S. cerevisiae*, is involved in implication of protein kinase C and glycerol pathway and is regulated by various phosphorylation sites present in it [32]. Hsp90 is an essential component of cytoplasmic Hsp90-Hsp70 chaperone network responsible for protein folding. Protein emerging from ribosome is initially folded in nascent polypeptide by Hsp70 and then passed to the Hsp90 machine which performs later folding [33]. Hsp90 maintains the integrity of client protein which on interaction modulates weak ATPase activity of Hsp90 followed by ATP hydrolysis and remodelling resulting in open form of protein [34]. Hsp70 and Hsp40 are cochaperons of Hsp90, which acts simultaneously. Hsp70 requires adapter protein (Sti1/Hop1) to associate with Hsp90 by inhibiting ATPase activity of Hsp90 which is before activated by Aha1 (Hsp90 cochaperone) ultimately causing polypeptide release [35]. Tah1/Pih1 (chromatin remodelling) and Sgt1 (contributes to kinetochore assembly) are the cochaperons of Hsp90 in* S. cerevisiae*. Tah1/Pih1 is involved in regulation of Hsp90 chaperone complex by inhibiting Hsp90 ATPase activity whereas Sgt1 has no effect on ATPase activity [36]. The other cochaperon Cdc37 with Hsp90 modulates function of crucial cell cycle regulator, Cdc28, Cdc50, Cdc60, and Swe1 [37]. Acetylation of K270 residue is important for Hsp90 function in* S. cerevisiae* [38].* S. cerevisiae* is known to have three small Hsps, that is, Hsp30, Hsp26, and Hsp12. Hsp30 is involved in energy conservation by inhibiting ATPase during stress conditions [20]. Mutation in Hsp70 leads to expression of* hsp* genes at temperatures that are optimal for growth of the organisms [39]. Msn2 and Msn4 (140 kDa) are the regulatory proteins which are involved in the activation and expression of* hsp12* by recognising stress response elements with characteristic nucleotide sequence “CCCCT” [20]. Regulatory factors of* S. cerevisiae*, Yap1, and Yap2 account for the regulation of Hsp30 [40]. For the promoters of* hsp70* and* hsp12* genes, GTPase of Ras family and cAMP act as negative control [41]. Transcription and posttranscription processes involve regulation of* hsp90*, regulated by Hsf1 in* S. cerevisiae *[42, 43]. Hsf1 (heat-shock transcription factor) is activated by hyper phosphorylation in response to heat-shock via heat-shock element that leads to increased transcription and accumulation of heat-shock gene products (Hsp60, Hsp70, Hsp78, Hsp90, and Hsp104) [42, 44]. Hsp70 interacts with Hsf1 encoding protein required for the function of Hsp90 involved in the repression of Hsf1 by feedback inhibition mechanism [45, 46]. MAPK (Slt2) acts as a client protein for Hsp90 which activates Rlm1 (transcriptional factor) involved in maintenance of cell integrity [47, 48]. Hsp12 maintains normal cell morphology that is essential for survival and growth [49].

In* C. albicans* morphogenesis is profoundly influenced by temperature and negatively regulated by Hsp90 by repressing Ras/PKA pathway, which is a positive regulator of morphogenesis in* C. albicans*. Thus, Hsp90 in* C. albicans* functions as a morphological controller [50]. At low temperature (11–15°C) Ras/PKA pathway is repressed by activation of Hsp90 which relieves Ras/PKA pathway at elevated temperature (37°C) resulting in filamentation [51, 52]. Hsp12 protein in cell wall is induced during stationary growth phase of morphogenesis and has a facilitating role in hyphal formation [53]. Hsp90 regulates Slt2 (stress-activated, mitogen-activated protein kinase) and Mkc1 (calcineurin), which are involved in maintaining cell integrity via MAPK pathway [54]. Other factors such as drugs and human steroid hormones influence the morphogenesis of* C. albicans* [55, 56].

Hsp90 protein has been studied in filamentous fungus* A. fumigatus* by Lamoth et al. [6]. Repression of* hsp90* gene showed decreased spore viability, decreased hyphal growth, and severe defects in germination and conidiation. Downregulation of the conidiation-specific transcription factors BrlA, WetA, and AbaA was reported [6]. Under heat stress, Hsp90 protein moves from cytosol to nucleus and carries nuclear localization signal suggesting that it might have role in transcriptional regulation during heat stress [6]. Thus, it suggests that Hsp90 holds a key role in morphogenesis of* A. fumigatus*.

### 2.1. Conidiation

Conidia are produced by asexual sporulation in filamentous fungi. They are generally produced after mycelial stage in vegetative growth [83]. In* Aspergillus* species (*Aspergillus nidulans* and* A. fumigatus*) conidiation is controlled by Hsp90-calcineurin pathway; deletion of calcineurin resulted in impaired hyphal growth, decrease in *β*-glucan content of cell wall, and defective sporulation. Downregulation of transcriptional factor (BrlA, WetA, and FlbA) in* A. nidulans* and (BrlA, FlbA, and AbaA) in* A. fumigatus* has been reported during inhibition of Hsp90-calcineurin pathway [6, 84, 85]. Hsp90 is widely distributed in cytosol under standard growth and moves to organs according to stress conditions such as in nucleus under heat stress and in cell wall or hyphal tips under cell wall stress [6]. Induction of Hsp90 has been shown to be essential during caspofungin drug treatment in* A. fumigatus* [86]. Reverse internal acetylation has been reported in* A. fumigatus *Hsp90, which is an important regulatory mechanism of Hsp90 [21].

Transcription factor (Hsf1) in* C. albicans* is temporarily activated during thermal stress (e.g., 37°C) [42]. The function of transcription factor, Hsf1, is repressed by Hsp90 which involves client proteins secretion, vesicular transport, and mitochondrial membrane components [87]. In dormant conidial stage of yeast (*N. crassa*,* C. albicans, *and* S. cerevisiae*), Hsp30-Hsp80 complex with Hsp70 interacts with unfolded polypeptide (homologous with Hsp90) [88, 89]. From the previous studies it has been shown that* hsp70* transcripts in* N. crassa* are predominant at aerial and dormant conidia stage which fluctuate on further progressive stages of germination [90]. The expression of* hsp70* transcripts increases during lag and log phase, declined in young aerial hyphae, and is maximum at late aerial hyphae due to transcriptional activation or may be due to decrease in rate of mRNA degradation [91].

### 2.2. Dimorphism

Dimorphism is the property of fungus in which it converts from one form to another (mycelia to yeast or vice versa) in response to various stress conditions and so plays a key factor in fungal virulence (e.g.,* P. lutzii*,* C. albicans*, and* H. capsulatum*) [92]. In normal growth conditions* Paracoccidioides* exists in mycelial stage and converts in hyphal stage but as the temperature increases (37°C) it converts into yeast form which is a pathogenic form.

*Paracoccidioides *species causes Paracoccidioidomycosis and is endemic to South America. The infection is predominant in male in comparison to female and 17*β*-estradiol has been reported to inhibit the transition from mycelia form (infective propagules) to yeast form [93, 94]. In yeast form of* P. brasiliensis* or* P. lutzii*,* hsp70* showed high transcripts [95]. During transition from mycelia to yeast form* hsp90* transcript was upregulated at early stage [93]. Thus, Hsps are important in the dimorphism and cell viability of* Paracoccidioides *[93, 96]. Calcineurin helps Hsp90 in maintaining environmental changes by regulating dimorphism but not proliferation [97]. The mRNA expression of* hsp90* is higher in yeast than mycelial form and gene expression is upregulated during early phase of mycelium to yeast transition. Hsp90 impairs yeast proliferation at 37°C but slightly affects mycelial proliferation. The transition from yeast to mycelia form in* Paracoccidioides* occurs independent of Hsp90 activity. Hsp70 protein is found to be expressed in the yeast phase [23]. Hsp60 is also upregulated in response to thermal stress and involved in differentiation, infection, and colonization [98, 99]. At mycelial stage Calnexin gene, a type of* hsp60* gene (cytoplasm), and* sba1* cochaperon gene were overexpressed whereas in yeast form gene encoding for cochaperons, for example,* cpr1*,* hsp42*,* hsp60*,* hsp70,* and* hsp90,* were upregulated. Thus, it suggested that expression of genes encoding for Hsps is more in yeast form of* P. brasiliensis* [100].

In yeast form of* C. albicans* Hsp90 plays a negative regulatory role in the conversion from yeast to filamentous form which is positively regulated by Ras/PKA pathway [52]. At an elevated temperature (37°C) filamentation of* C. albicans* occur in serum leading to virulence. Thus, it suggests that repression of Hsp90 is responsible for inducing yeast to filamentous form. Hsp12p affected by quorum sensing molecule, farnesol, blocks yeast to hyphal transition via cAMP dependent signalling cascade [61]. Surface invasion Ssa (Hsp70) in* C*.* albicans* has been reported to be upregulated at hyphal stage [92].

## 3. Role of Hsp in Stress Tolerance

### 3.1. Temperature

Various problems caused by temperature change are associated with the temperature dependent morphological transitions and protein folding [101]. Heat-shock response is generally seen in the cells affected by thermal stress [12]. In several studies it has been reported that reactive oxygen species (ROS) production is enhanced in cells under thermal stress which also activates Hsps [102]. Temperature stress in dimorphic fungus may have an effect on different phases of life cycle, so protein expression may be phase specific or heat induced [103].

In a model organism* N. crassa*, at temperature 25–37°C, mycelial form converts to hyphal form with the expression of 70 kDa Hsp [104]. As temperature increases (e.g., 45°C), the mycelial form converts to yeast form in* N. crassa*; the major Hsps of 67, 83, and 98 kDa and minor Hsps of 30 kDa were expressed at germinating conidiophore stage which showed normal growth after one hour [105]. As it has been suggested, upregulated Hsp90 protein and their interaction with calcineurin are responsible for mycelia to yeast transition in* C albicans*,* S. cerevisiae,* and* P. brasiliensis*; low level of Hsp90 causes reduction in expression of calcineurin catalytic subunit (CNA2) [97]. As we discussed, in* N. crassa,* Hsp90 inhibition is responsible for yeast to filamentous transition which is antagonist to Ras/PKA pathway. Thus, it is suggestive that Ras/PKA pathway positively regulates yeast to filament transition and is negatively regulated by Hsp90 [106]. In* A. fumigatus *Hsp90 is involved in initiation of germination and hyphal elongation of dormant conidia [86].

Hsp90 in* P. brasiliensis* prevents cellular and molecular damage of cells in response to heat stress. It plays a role by regulating the level of ROS. At elevated temperature (42°C) for 4 hr, ROS level was found to be increased on inhibition of Hsp90 in yeast cells and showed no effect on ROS level at optimum temperature (37°C). This study suggested that Hsp90 regulates ROS level only in heat stress [97].* hsp60* has increased level of mRNA expression in heat stress conditions. There is a 5.9–6.9-fold increase in* hsp60* mRNA expression that was observed in* A. fumigatus* and* Aspergillus terreus* at 40°C [13]. In a limited data set of expressed sequence tag analyses of* A. fumigatus* derived at 37°C did not observe gene encoding for Hsps [4, 107].* Trichophyton mentagrophyte* showed 4.9-fold increased expression of* hsp60* when incubated at 40°C [13].* C. albicans* showed 3.2-fold upregulation at 30°C. So it can be said that* hsp60* induction was highest at 35°C to 40°C, which depends on types of fungus. In* Cladosporium cladosporioides* no* hsp60* transcripts were observed at low optimum temperature (20°C) for growth [13]. Hsp60 plays an important role in fungal related diseases in humans and acts as an immunological trigger and increase in fungal* hsp60* mRNA has been reported [13, 108].

At high temperature, Hsp104 protein stabilizes stationary-phase yeast cells and aerobically growing cells of* S. cerevisiae* [19, 109]. Further, at high temperature, Hsp104 is involved in unfolding of denatured protein with the help of Hsp40 and Hsp70 [29]. Deletion of the* hsp*104 gene results in the loss of tolerance to not only heat but also the viability of cells stored at low temperatures [20]. On functional basis, Hsp104 showed similarity with Hsp70, which works alternatively in response to thermotolerance [110]. Overexpressed* hsp12* also contributes to temperature resistance in* S. cerevisiae* by accumulating the trehalose [111]. However, in a recent study, it has been shown that trehalose-6P synthase (Tps1) protein is essential but not trehalose in yeast to maintain the ATP requirement in a heat-shock condition [112].

Hsp30 level also increases at the time of thermotolerance in* S. cerevisiae*. Heat stress results in increase in membrane fluidity which is controlled by increase in amount of Hsp30 in membrane [113]. Heat stress also increases Hsp10 (cochaperone) and Hsp78 (chaperone) in mitochondrial matrix which inhibits Hsp60 ATPase activity resulting in protein folding in* S. cerevisiae* [113, 114].

Hsps also play an important role in response to freezing temperature in* S. cerevisiae*. Recent studies showed that when yeast cells were stored at −20°C, there is an increase in resistance against low temperature [115]. This was due to the expression of Hsp12, which suggested that Hsp12 plays a role in freeze tolerance. Sometimes, the role of Hsp12 is interchangeable with trehalose. Hsp12 protein expression is induced at 4°C and 0°C, important for adaptation to cold in* S. cerevisiae* [115, 116]. So, Hsp12 plays an important role in cryopreservation to maintain viability of cells. Hsp12 shows similarity with trehalose activity in maintaining membrane integrity against desiccation [117]. Hsp12 functions at plasma membrane level to maintain cells integrity in freezing stage in* S. cerevisiae* [111].

### 3.2. pH

pH plays important role in* hsp* gene expression which involves PacC/PalA pathway. The PalA protein is a member of conserved signalling cascade and is involved in pH mediated regulation of gene expression in* A. nidulans*. PacC is a regulator which is required for activation/repression of acid/alkaline associated gene in filamentous fungi [118, 119]. The PalA protein mediates the proteolytic activation of PacC [120]. Freitas et al. showed that at optimum temperature and acidic pH,* hsp30*,* hsp70,* and* hsp90 *genes are induced depending upon the extracellular pH conditions. In mycelial culture of* A. nidulans* at pH 5.0, transcript level of* hsp30, hsp70*, and* hsp90 *is preferentially high. At alkaline pH, in the presence of PalA environment, there is a decrease in* hsp30 *transcripts that has been observed.* hsp70* transcripts were observed to be high at pH 8 in PalA environment. Also, change of pH (alkaline or acidic) does not influence the transcription of* hsp90 *in* A. nidulans* [118]. Generally, oxidative and osmotic stresses and heat-shock cause an increase in transient transcription rate in yeast in comparison to high alkaline pH stress [121].

### 3.3. Osmotic Pressure

Osmotic pressure is responsible for causing membrane destabilization by increasing membrane fluidity which is overcome by induction of Hsp12 and Hsp26 [122].* hsp*12 and* hsp*26 genes are generally regulated by Msn2/Msn4p (Trans-activators), which helps in activation under pressure stress [123]. Small Hsp26p has molecular chaperone activity help membrane from irreversible aggregation of proteins [69]. Hsp30, a plasma membrane protein, is also found upregulated in yeast cells under pressure related stress conditions but independent of Msn2/Msn4p [40]. Hsp30 works by downregulating the activity of H^+^ ATPase on ATP depletion and so plays a role in energy conservation [113, 124]. Hsp31 acts as a molecular chaperone in response to pressure stress. It is generally present in endoplasmic reticulum and plays partial role in growth and catalysis of misfolded proteins at 25 MPa [113]. The loss of mitochondrial function due to pressure stress is overcome by upregulation of Hsp60 and Hsp78 [125]. At high pressure conditions (150–180 MPa), induction of Hsp104 takes place, leads to unfolding of denatured proteins, and increases viability of cells [126, 127].

## 4. Heat-Shock Protein as Antifungal Targets

Toxicity always remained a serious concern and a drug target is an emerging area. In current scenario to overcome various fungal related diseases in both plants and animals, research is being focused on the development of various therapeutic targets. One such interesting area to focus on is Hsps, due to its very wide role in fungal survival during stress conditions. In recent years, Hsp90 has been proposed as antifungal target. Hsp90 inhibitors geldanamycin and their derivatives showed antifungal activity and synergistic effect with caspofungin against* A. fumigatus* and* C. albicans* [86, 128, 129].

In* P. lutzii, P. brasiliensis *morphological transitions (mycelia to yeast) are important for causing disease, which involves the role of Hsp90, as Hsp90 strengthens stress response in these fungi [97]. When these* Paracoccidioides* species were treated with benzoquinone ansamycin antibiotic and geldanamycin (inhibitor of Hsp90), inhibition from mycelial to yeast transition was observed and causes the diminishing of yeast form. Hsps have also important role in drug resistance. Hsp90 is involved in resisting fungi with the effect of azoles. In recent studies it has been seen that when fungi (*C. albicans, S. cerevisiae, *and* A. fumigatus*) have impairment or scarcity of Hsp90 then they are prone to antifungal drug, fluconazole (Azole family), while in the presence of Hsp90 they showed resistance to fluconazole effect. Hsp90 develops the resistance property which involves calcineurin pathway [130]. Activation of Hsp90-calcineurin complex leads to drug resistance (Geldanamycin) and various stress responses. Complete inhibition of Hsp90 is difficult as it is highly expressed in basal conditions and highly conserved [86]. Hsp70 have been found to modulate the effect of caspofungin via Hsp90-Hop/Sti1 (cochaperons) in* A. fumigatus*. Hsp70 have Sti1 binding region which on activation responds to thermal stress and caspofungin effect. Hsp104 have ATPase activity and play an important role in stress response via activating Hsp70 and so help in protein refolding of aggregated proteins. Being involved in such important function, Hsp104 also emerged as important antifungal target. However, in a genome wide gene expression study of* A. fumigatus* in response to amphotericin B showed downregulation of* hsp88* transcripts [131]. Studies on* S. cerevisiae* showed that treatment of small amount of Guanidinium hydrochloride with yeast inhibits the activity of Hsp104 by binding the M-domain of Hsp104, hence inhibiting the Hsp104-Hsp70 interaction, causing the inhibition of stress tolerance of yeast [132].

Due to unavailability of commercial fungal vaccines for humans, it is a matter of great significance to develop vaccines against fungal infections. This is the issue of challenge in both scientific and technological aspect because of the lack of understanding of immune response against fungal antigens. Recent studies showed that heat killed yeast can be used as vaccine against five different fungal infections. Antigen having the same antigenic epitope in different fungi is potentially recognised by the same antibodies against them, which could inhibit fungal growth and development [133]. Various Hsps such as Hsp70, Hsp40, Hsp90, and Hsp60 has been shown to be upregulated when different sets of fungi (*C. albicans, P. brasiliensis, *and* Coccidioides posadasii*) were injected in mice model [134]. Hsps come under conserved protein domain common in fungi which commonly act as an antigen in fungal infections and showed potential for the development of pan fungal vaccine, providing absence of antigenic region of fungal Hsps in human counterpart.

## 5. Conclusion

Heat-shock proteins are expressed during various stress conditions. Expression of Hsps may be specific to different conditions, for example, temperature, osmotic pressure, pH, antifungals, and oxidative stress. Hsp90 and Hsp70 are the predominant Hsps found in the morphogenesis of fungi. They work individually or in Hsp90-Hsp70 complex in different fungi and play an important role in nascent folding of aggregated polypeptide, hence providing stability. Hsp40 and Hsp104 are also found to be upregulated during morphogenesis of fungi. In dimorphism expression of various Hsps (Hsp90, Hsp70, Hsp60, and Hsp40) is unregulated in the yeast stage. So we can conclude that in yeast form expression of Hsp is higher than mycelial stage and involves Ras/PKA pathway. Heat stress induces the expression of various Hsps (Hsp90 and Hsp60), whereas Hsp12 is found to be upregulated in freeze tolerance of fungi. Fungus activates PacC/PalA pathway to withstand pH stress. In the presence of* palA gene*, Hsp30, Hsp70, and Hsp90 are predominantly upregulated in acidic pH, but level of Hsp30 decreases in alkaline pH. Osmotic stress response is overcome by upregulating Hsp12 and Hsp26 in membrane, Hsp78 and Hsp60 in mitochondria, Hsp30 in plasma membrane, and Hsp31 in endoplasmic reticulum. Hsp104 is expressed at high hydrostatic pressure. Hsp70, Hsp90, and Hsp104 are found to be expressed in response to various antifungal compounds, so these Hsps can be studied further as antifungal targets. Thus further study of fungal Hsps at mRNA level and protein level needs to be investigated to understand the biology of organism and to develop potent antifungal targets to overcome fungal related losses.

## Figures and Tables

**Table 1:** Description of heat-shock protein in fungi based on molecular weight, cellular location, and their functions.

Hsps	Molecular weight (kDa)	Cellular location	Cellular functions	References
Hsp8.5	8	CyP	Ubiquitination	[57, 58]

Hsp10	10	M	Stabilize catalytic subunit of DNA polymerase-*α* and protein folding in mitochondria	[59, 60]

Hsp12	10–20	CyP, CW, PM	Stress tolerance, maintaining cell morphology, cell adhesion, and germ tube formation	[49, 61]

Hsp17	10–20	M	Membrane lipid bilayer stabilizer	[57]

Hsp21	20–30	CW, ER	Fungal adaptation in environmental stress and pathogenicity, glycerol and glycogen regulation, virulence factor in eukaryotic pathogens, and hyphal formation	[62, 63]

Hsp26	20–30	CyP	Induced in low pH conditions	[28, 64]

Hsp27	20–30	N, CyP	Cytoskeleton	[57]

Hsp30	30	PM	Regulates membrane function under heat shock conditions, negatively regulates H^+^ ATPase	[40]

Hsp31	30	ER	Growth under partial pressure conditions and act as molecular chaperone	[65]

Hsp32	30	CyP	Heme-oxygenase	[57, 63]

Hsp40	40	CyS, M, ER	Cell physiology and cofactor of Hsp70	[66, 67]

Hsp42	40	CyS	Suppress the aggregation of nonnative protein	[68]

Hsp60	60	M, CyS	Immunological properties, upregulated in biotic and abiotic stress	[69–71]

Hsp70	68	CyS, N, ER, R, M	Initial folding of nascent polypeptide and ATPase activity	[34, 72, 73]

Hsp78	70	M	Mitochondrial thermotolerance and pressure tolerance	[73]

Hsp80	80	CyP	Interact with unfolded polypeptide individually or in complex	[73]

Hsp82	80	CyP	Pheromone signalling and negative regulation of Hsf1	[73, 74]

Hsp83	83	CyS	Interaction with nascent chain polypeptide and signal transduction	[24]

Hsp88	88	CyS	Interact with Hsp30	[75, 76]

Hsp90	90	CyS, ER, N	Folding and maintenance of client proteins, involved in transcriptional and posttranscriptional processes and activation of signal transducers	[72, 77]

Hsp100	100	CyS	Catalytic activity and protease with ATPase activity	[20]

Hsp104	100	CyS	Thermotolerance, survival at stationary phase, ethanol tolerance, reactivate denatured and aggregated proteins, and replication of yeast prions	[29, 78, 79]

Hsp105	105	N, CyP	Not reported	[57, 80]

Hsp110	110	N, CyP	Misfolding of polypeptide and hydrolyses of ATP	[57, 81, 82]

CyP: cytoplasm; CyS: cytosol; M: mitochondria; PM: plasma membrane; ER: endoplasmic reticulum; N: nucleus.
